# Vestibulo-Hippocampal Function Is Enhanced and Brain Structure Altered in Professional Ballet Dancers

**DOI:** 10.3389/fnint.2018.00050

**Published:** 2018-10-18

**Authors:** Milos Dordevic, Robert Schrader, Marco Taubert, Patrick Müller, Anita Hökelmann, Notger G. Müller

**Affiliations:** ^1^German Center for Neurodegenerative Diseases (DZNE), Magdeburg, Germany; ^2^Neurology Clinic, Otto von Guericke University Magdeburg, Magdeburg, Germany; ^3^Institute of Sports Science, Otto von Guericke University Magdeburg, Magdeburg, Germany; ^4^Center for Behavioral Brain Sciences (CBBS), Magdeburg, Germany

**Keywords:** balance, ballet, orientation, vestibular system, hippocampus, MRI, VBM

## Abstract

**Background and Objective:** Life-long balance training has been shown to affect brain structure, including the hippocampus. Data are missing in this respect on professional ballet dancers of both genders. It is also unknown whether transfer effects exist on general balancing as well as spatial orientation abilities, a function mainly supported by the hippocampus. We aimed to assess differences in gray matter (GM) structure, general balancing skills, and spatial orientation skills between professional ballet dancers and non-dancers.

**Methods:** Nineteen professional ballet dancers aged 18–35 (27.5 ± 4.1 years; 10 females) and nineteen age-matched non-dancers (26.5 ± 2.1 years; 10 females) were investigated. Main outcomes assessed were the score of a 30-item clinical balance test (CBT), the average error distance (in centimeters) on triangle completion task, and difference in GM density as seen by voxel-based morphometric analysis (VBM, SPM).

**Results:** Ballet group performed significantly better on all conditions of the CBT and in the wheelchair (vestibular-dependent) condition of the spatial orientation test. Larger GM volumes for ballet dancers were observed in the right hippocampus, parahippocampal gyrus, insula, and cingulate motor cortex, whereas both larger and smaller volumes were detected within cerebellum bilaterally in comparison to non-dancers.

**Conclusion:** Our results indicate that life-long ballet training could lead to better clinically relevant balancing abilities as well as vestibular-dependent spatial orientation capabilities; both of the benefits might be caused by positive influence of ballet training on the vestibular system function, and—possibly—its connectivity with temporal lobe regions responsible for vestibular-dependent orienting in space.

## Introduction

Ballet dancing does not only require the coordination of complex movement patterns but also demanding in terms of the processing of vestibular input and maintaining balance. The assumption of a vestibulo-hippocampal dependency is supported by previous research in humans which showed that complete abolition of vestibular input, due to bilateral vestibulectomy, leads to atrophy in distinct medial temporal lobe areas (Brandt et al., 2005). Results of more recent studies also revealed hippocampal changes in patients suffering from various vestibular system disorders (Zu Eulenburg et al., 2010; Göttlich et al., 2016; Kremmyda et al., 2016). Multiple pathways that exist between the two have been discovered (Hitier et al., 2014), and there is a disruption in function of the latter when the input from the former ceases (Stackman et al., 2002; Russell et al., 2003). One study on professional ballet dancers, together with slackliners and ice-skaters, has demonstrated structural differences in temporal brain regions, particularly the hippocampus, compared to normal controls (Hüfner et al., 2011). This study could not, however, answer the question on neuroanatomical differences specifically related to ballet dancing, since it included other professionals in its cohort. Another study reported decrements in gray matter (GM) of several brain regions (Hänggi et al., 2010), including both cortical and subcortical structures, but the cohort consisted of only female ballet dancers, whose brain and development have already been shown to differ from that of males (Giedd et al., 1999; Good et al., 2001). Therefore, based on previous research it was not possible to determine which specific brain regions show significant differences in GM when comparing professional ballet dancers to non-dancers.

Another remaining question pertains to non-visual-dependent spatial orientation skills, as well as balancing skills, of ballet dancers. That is, it is unknown if these professionals have better developed non-visual-dependent abilities to orientate in space and balance when compared to persons not involved in such activities. Results of our own previously published research indicated that one-month of intensive slackline training can lead to significant improvements in these abilities (Dordevic et al., 2017b). The eventual findings in this respect could also be useful for a better understanding of both healthy aging and dementia prevention, as the decline in this ability has been linked to the degeneration of spatial navigation centers, located in the hippocampus and surrounding temporal brain areas (Allen et al., 2004). Additionally, loss of balance is an important cause of injuries, especially in old age, and it represents a major burden for the health system (Carter et al., 2001; Kannus et al., 2005; Sherrington et al., 2011).

Accordingly we hypothesized that: (1) brain regions, and particularly those responsible for balance and spatial orientation functions, of professional ballet dancers would show different structure compared to non-dancers and (2) ballet dancers would perform better in balancing and spatial orientation tasks, especially those that are vestibular-dependent (in which the vestibular input is the dominant one and thus governs the performance).

Hence, the goal of our study was to clarify the differences between professional ballet dancers and the normal population with regards to brain structure, non-visual spatial orientation abilities, and general balancing abilities.

## Materials and Methods

### Ethical Approval

This study was carried out in accordance with the recommendations of guidelines of Ethics Committee of the Medical Faculty at the Otto von Guericke University (approval number: 156/14) with written informed consent from all subjects. All subjects gave written informed consent in accordance with the Declaration of Helsinki. The protocol was approved by the Ethics Committee of the Medical Faculty at the Otto von Guericke University.

### Subjects

Twenty-six professional ballet dancers (18–35 years old) were initially recruited for this study. Since seven of them were not eligible for MRI scanning according to the safety procedures of our department (tooth braces and similar metal implants, tinnitus, etc.), they had to be excluded. The remaining nineteen ballet dancers were age- and gender-matched by the control participants (**Table 1**). Physical activity was assessed by asking subjects how many hours they spend on training weekly on average; all sports were taken into consideration, including jogging, various team sports, cycling, etc., but not walking. Participants of both groups were paid the same amount of money for their participation in the study. Sample size and characteristics, as well as the balance-training duration have been justified by our previous studies (Dordevic et al., 2017a,b).

**Table 1:** Test conditions of the Clinical balance test (CBT).

No.	Condition	Task		Points (min = 0, max = 3)			
				0	1	2	3
1	Static–stable surface (floor)	Stand with feet together–open eyes					
2		Stand with feet together–closed eyes					
3		One leg stance–left–open eyes					
4		One leg stance–right–open eyes					
5		One leg stance–left–closed eyes					
6		One leg stance–right–closed eyes					
7	Static–unstable surface (pad)	Stand normally (hip width stance)–open eyes					
8		Stand with feet together–open eyes					
9		Stand normally (hip width stance)–closed eyes					
10		Stand with feet together–closed eyes					
11		One leg stance–left–open eyes					
12		One leg stance–right–open eyes					
13		One leg stance–left–closed eyes					
14		One leg stance–right–closed eyes					
15	Dynamic	Walk inside the zone (4 m × 30 cm)	Forward				
16			Turn (90°)				
17			Backward				
18		Walk on the line (4 m × 5 cm)	Forward				
19			Turn (90°)				
20			Backward				
21		Walk on the line with feet one after the other (4 m × 5 cm)	Forward				
22			Turn (90°)				
23			Backward				
24		Walk on the beam (4 m × 10 cm)	Forward				
25			Turn (90°)				
26			Backward				
27		Walk on the beam sideways (4 m × 10 cm)	Rightward				
28			Turn (90°)				
29			Leftward				
30		Walk on the line with closed eyes (4 m × 5 cm)	Forward				


Eligible control subjects for this study were all those aged from 18 to 35 years who had no previous experience in any ballet-related similar activity (i.e., highly demanding balancing activities, such as slacklining, rhythmic gymnastics, etc.) and had normal or corrected to normal vision. Exclusion criteria were injuries to the musculoskeletal system and systemic diseases (e.g., cardiovascular, metabolic, nervous system diseases, etc.) that might influence their performance. Participants were recruited through advertisement in the buildings of Otto von Guericke University in Magdeburg.

### Study Design

This study was planned and organized as a cross-sectional one with one factor—namely group (control, ballet). The participants of the control group were age- and gender-matched to the participants of the ballet group. All the measurements took place in the movement lab of the German Center for Neurodegenerative Diseases from July 2015 to December 2016.

### Behavioral Tests

Both tests have been described in our previously published work (Dordevic et al., 2017a). In brief, clinical balance test (CBT) consisted of standing on stable and unstable surfaces and walking conditions, all of which further contain sub-conditions with open and closed eyes.

Standing conditions included:

• Two- and one-leg stance on both stable (floor) and unstable (soft pad) surfaces, with both open and closed eyes.

Walking conditions included:

• Walking forward, backward, and turning inside a 30 cm wide and 4 m long polygon with open eyes, followed by the same test on a 5 cm wide line as well as on 10 cm wide balance beam.
• Walking forward on 5 cm line with closed eyes.

In total, there are 30 assessment items within this test, 14 of which assess standing; 16 walking; and 8 of all measurements are performed with closed eyes. The maximum amount of points that could be collected on the test was 90, with each condition carrying the minimum of 0 and the maximum of 3 points. In each of the standing conditions participants were instructed to maintain the required position for 15 s, whereas in walking conditions there was no time requirement and participants were asked to walk at their own pace. For detailed list of conditions see **Table 1**.

For assessment of non-visual spatial orientation the triangle completion test (TCT) was used. In brief, six triangular paths were marked on the floor of a room, three in the left and three in the right direction, giving thus three pairs of triangular paths, with turning angles of 60, 90, and 120°. The test consisted of two conditions: active-walking and passive-wheelchair. In the active-walking condition, while being guided on foot, the participant’s movement was controlled by leading him or her along two sides of the triangular path as he or she held onto a wooden bar. The passive-wheelchair condition included transport along the same routes with the use of a standard wheelchair with attached footpads. Each participant was walked (active) and pushed (passive) only once along each of the paths, giving thus 12 trials per participant in total (3 to the left and 3 to the right, times 2 conditions). Once the participant was walked/pushed in the wheelchair along two sides of each triangle, his or her task was to walk along the third one, back to the starting point, using thus the shortest possible way back; that is, the participants were instructed not to walk back along the two sides that were used to bring them to the drop-off point, but to use the shortest possible way back to the starting point instead, which is actually always the third side of the respective triangle. The first main outcome variables were the distance error on each trial, which was assessed by marking the participant’s stopping point with adhesive dots on the floor and later measuring the distance from that stopping point to the starting point, from which the respective movement was initiated. The second outcome variable was angular error, which was estimated as the angular deviation from the optimal direction (that would take directly to the start point). For the whole duration of the test participants were blindfolded in a quiet room and thereby could not use any visual or auditory cues that might help them in finding their way back to the starting point. It can thus be assumed that the only cues they could use were somatosensory and vestibular in the active-walking condition and vestibular only in the passive-wheelchair condition.

### MRI

MR images were acquired on a 3 Tesla Siemens MAGNETOM Verio scanner (Syngo MR B17) using a 32-channel head coil. High-resolution T1-weighted MPRAGE sequences were acquired using a 3D magnetization-prepared rapid gradient echo imaging protocol (224 sagittal slices, voxel size: 0.8 mm × 0.8 mm × 0.8 mm, TR: 2,500 ms, TE: 3.47 ms, TI: 1,100 ms, and flip angle: 7°).

Voxel-based morphometry (VBM) is a whole-brain unbiased technique for analysis of regional GM volume and tissue changes (Ashburner and Friston, 2000). Preprocessing involved gray-matter segmentation, template creation *via* DARTEL, spatial normalization to standardized Montreal Neurological Institute (MNI) space and smoothing with an Gaussian kernel of 8 mm full width at half maximum (FWHM).

### Outcome Variables and Data Analysis

The outcome variable for the neuroanatomical analysis was the structural difference in brain neuroanatomy as observed by VBM. In order to analyze the difference in GM volume changes between groups, an independent *t*-test with the factor group (ballet, control) was applied. Since the whole-brain between-group comparison was carried out, multiple comparison correction was also performed in the form of Family-wise-error (FWE) correction, where the results were considered significant at *p* < 0.05, unless otherwise specified (uncorrected *p* < 0.001). Data were analyzed with MatLab (Mathworks, United States) and SPM12 (UCL, Great Britain). The results of the VBM analyses are visualized using the xjView toolbox^1^.

Analysis of the behavioral data was performed with SPSS v.21 (IBM, United States), with the group (control, ballet) as factor. Independent *t*-test was run after checking for assumptions of normality and homogeneity of variance. If the assumptions were not met, the non-parametric equivalent (Mann–Whitney *U*-test) was applied.

In tables and figures the respective means with 95% confidence intervals of the difference are presented. In addition the effect sizes are calculated and listed.

## Results

The final analysis included 19 participants in each group. All subjects were recruited from July 2015 to December 2016 and their characteristics are shown in **Table 2**. The participants of the two groups did significantly differ in weight (*p* = 0.02) and amount of training hours per week (*p* < 0.001), whereas in other characteristics there was no significant difference.

**Table 2:** Characteristics of participants.

Characteristic	Training (*n* = 19)	Control (*n* = 19)
Age (years)	27.5 ± 4.1	26.5 ± 2.1
Age when training begun (min–max)	8.0 ± 3.8 (3–16)	–
Sex (females)	10 (53%)	10 (53%)
Weight (kg)	59.4 ± 11.6	67.9 ± 10.4
Height (cm)	169.3 ± 10.1	172.5 ± 8.4
Hours of activity–per week	33.4 ± 13.5	3.3 ± 1.6
Handedness–right	18 (95%)	19 (100%)
Ethnic origin		
• European	16 (84%)	17 (90%)
• Asian (Indian)	0 (0%)	2 (10%)
• Asian (Japanese)	3 (16%)	0 (0%)


### Behavioral Tests

**FIGURE 1:**
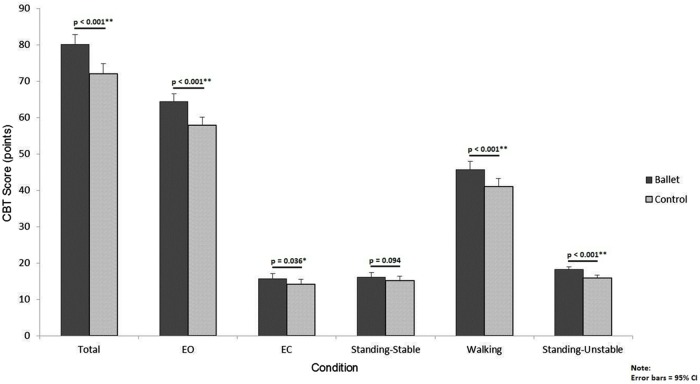
Comparison of ballet and control groups on all conditions of the clinical balance test (CBT).

As illustrated in the **Figure 1**, the ballet dancers performed significantly better on the CBT, which was true for all sub-conditions of the test except for the simplest task which involved standing on stable flat surface. In the **Table 3** are listed the mean values for the two groups as well as the effect sizes and confidence intervals of the difference between the two groups. The effect size was large to very large for all comparisons, including the condition where no significant difference was observed.

**Table 3:** Comparison of ballet and control groups on all conditions of the clinical balance test (CBT).

Test (assessment scale)	Condition	Mean		95% CI of the difference	Effect size (d)
		Ballet	Control		
CBT (points)	Total	80.1	72.0	5.3 to 10.9	1.89
	Eyes open	64.4	57.9	4.3 to 8.6	1.98
	Eyes closed	15.7	14.1	0.1 to 3.1	0.70
	Standing-stable	16.2	15.1	-0.2 to 2.3	0.56
	Walking	45.7	41.1	2.5 to 6.8	1.43
	Standing-unstable	18.2	15.8	1.6 to 3.2	1.94
TCT (degrees)	Total	15.7	20.9	-8.2 to -2.1	0.22
	Walk	14.9	18.4	-7.7 to 0.5	0.08
	Wheelchair	16.6	23.3	-11.2 to -2.2	0.37
TCT (centimeters)	Total	106.9	122.5	-28.8 to 2.5	0.31
	Walk	103.2	108.9	-24.3 to 13.1	0.23
	Wheelchair	110.5	136.1	-44.0 to -7.4	0.39


**FIGURE 2:**
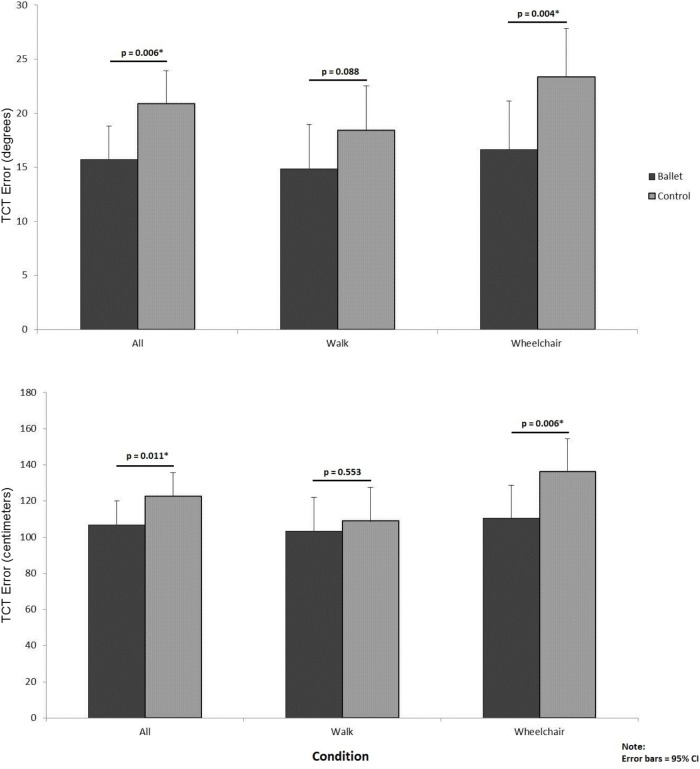
Comparison of ballet and control groups on both conditions of the triangle completion task (TCT).

The **Figure 2** illustrates the difference between the two groups on the TCT. The results demonstrated that ballet dancers performed significantly better on this test, by having smaller error in both distance and angle, which was mainly attributable to their better performance on the wheelchair (vestibular) condition. The results in the **Table 3** depict medium effect sizes for this condition.

### VBM Analysis

**FIGURE 3:**
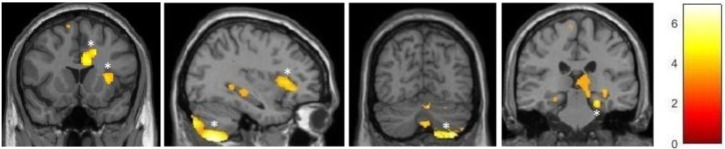
VBM observed GM increments in the ballet group compared to the control group. ^∗^FWE-corrected at the cluster level.

For the ballet group, the VBM analysis revealed significantly larger cluster-based FWE-corrected gray matter volumes within the inferior and posterior areas of the right cerebellar hemisphere, right parahippocampus, right cingulate motor cortex, and right insula (**Figure 3**). Additional tendencies at uncorrected level (*p* < 0.001) could be observed in the vermis, right posterior hippocampus, and right posterior thalamus. The respective MNI coordinates as well as the cluster sizes are listed in the **Table 4**.

**Table 4:** MNI coordinates of VBM-detected gray matter changes in the ballet group as compared to the control group.

Brain region	Location	Direction of difference	MNI coordinates (*x, y*, and *z*)	Cluster size (in voxels)
Temporal	Right hippocampus	Larger	30, -26, -6	128
	Right parahippocampal gyrus	Larger	21, -32, -20	2,013
Temporo-parietal	Right insula	Larger	38, 24, 3	1,020
			27, 21, 2	
Cingulate cortex	Right hemisphere	Larger	6, 17, 24	800
			15, 17, 36	
Cerebellum	Right hemisphere	Larger	32, -68, -59	3,897
	Vermis	Larger	2, -68, -47	283
	Right hemisphere	Smaller	12, -65, -29	460
	Left hemisphere	Smaller	-12, -63, -29	30


**FIGURE 4:**
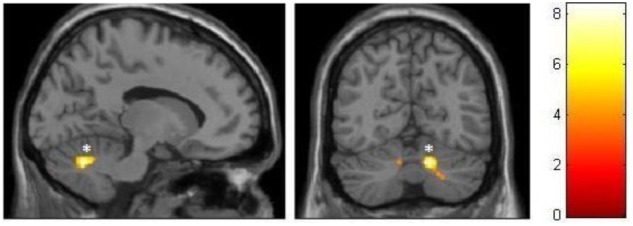
VBM observed GM decrements in the ballet group compared to the control group. ^∗^FWE corrected.

The control group participants also had significantly larger volume (FWE-corrected at cluster and voxel levels) within the cerebellum when compared to the ballet group, which was located within the right anterior lobe (**Figure 4**). A small tendency could also be observed at a similar location within the left cerebellar hemisphere. The respective MNI coordinates and sizes of these clusters are also presented in the **Table 4**.

## Discussion

The results of our study confirm both of our *a priori* hypotheses. That is, ballet dancers do have larger GM volumes in regions that contribute to balance and spatial orientation abilities, such as the posterior cerebellum and the vermis, insula, and hippocampal and parahippocampal regions, when compared to non-dancers; effects in the opposite direction, i.e., smaller GM volumes, were found in the cerebellar anterior lobes. On a behavioral level, in comparison with non-dancers, they demonstrated an increased ability to maintain balance in all conditions, as well as to orientate in space with closed eyes, especially in the mere vestibular-dependent condition, in which the blindfolded subjects did not walk themselves but were pushed in a wheelchair.

To date, to the best of our knowledge, there has been only one similar study on structural brain alterations in professional ballet dancers, however, this study only investigated females, and did not assess their spatial orientation and general balancing abilities, which prevents us from effectively comparing these with our findings. In the named study, Hänggi and colleagues (Hänggi et al., 2010) did not report any GM increments in ballet dancers compared to non-dancers. Instead they observed smaller volumes in several areas, including the supplementary motor and premotor areas and the putamen, all of which were located in the left hemisphere. This is in contrast to our findings, both regarding the localization and the direction of the effects. That is, apart from smaller GM volumes in the right and left cerebellar anterior lobes, we observed increments in the right posterior cerebellar hemisphere, vermis, right hippocampal and parahippocampal areas, and right posterior thalamus. This dissimilarity could be perhaps attributed to some of the cohort or methodological differences, including the sample, which in our case consisted of both genders, together with a higher mean age and the duration of professional activity of participants, as well as the VBM analysis procedure. For instance, the cerebellum and the pons are larger in men than in women and the difference is especially pronounced in the cerebellar hemispheres and the anterior vermis (Raz et al., 2001). Additionally, it has been shown not only that GM demonstrates a non-linear change during pre- and post-adolescence, but also that these developmental curves are not the same for all brain regions, with frontal lobe and parietal lobes peaking at the age of 12, the temporal at the age of 16, and the occipital only at the age of 20 (Giedd et al., 1999). Thus, the large discrepancies between the two studies may call for an additional study with a larger cohort so the structural brain differences between professional ballet dancers and non-dancers could be better delineated.

In an earlier study, we were able to detect some tendencies toward posterior hippocampal changes in a group of young healthy adults that had learned to slackline (Dordevic et al., submitted). The temporal dynamics of these brain changes partly resembled the balancing and spatial orientation skill levels of the participants which also showed a transient improvement. Increments in the posterior hippocampus have also been reported previously in ballet dancers by Hüffner and colleagues (Hüfner et al., 2011). Posterior hippocampal structural changes were also observed after year-long experience in taxi-driving and several sports with very high balancing demands, such as ballet dance, ice-skating, and slacklining (Maguire et al., 2000; Hüfner et al., 2011). Our sample of ballet dancers revealed similar results, indicating that long-term training might be necessary for structural changes in posterior hippocampus to persist. We could also demonstrate significant differences in the parahippocampal region between the ballet dancers and non-dancers. These were mainly located in the entorhinal cortex, particularly the medial entorhinal cortex. The entorhinal cortex is an interface between the three-layered hippocampal cortex and the six-layered neocortex and it provides the main cortical input to the hippocampus, with many reciprocal connections. More recently, an investigation of the medial entorhinal cortex (MEC) in animals, led to the discovery of grid cells that fire when the animal is in any of multiple locations that form a triangular grid (Moser et al., 2008, 2014; Sanders et al., 2015). Here the change in position can be computed based on vestibular information, sensorimotor information about self-motion, and optic flow. In our study on healthy older adults an increase in GM within parahippocampal regions was observed following an 18-month dance intervention (Müller et al., 2017). It is thus plausible to speculate that the better performance of ballet dancers in the TCT relies on these particular biological mechanisms, as well as that their long-term utilization causes neuroanatomical alterations which we were able to detect with VBM.

We observed highly significant differences in the caudal part of the cingulate cortex, area 24 of Broadman’s classification. It is known from functional MRI studies that the cingulate motor cortex and the cerebellum are active during interlimb coordinative movements, together with primary and associative sensory and motor regions of the cortex (Debaere et al., 2001). Somatotopy similar to that of the larger sensory-motor cortices can be found in those cingulate cortex regions where the changes were detected (Vogt and Pandya, 1987; Vogt et al., 1992; Paus et al., 1993; Petit et al., 1993; Wu et al., 2000). Since such coordinative movements are a core element of ballet dancing, the finding of a larger volume of this brain region in dancers makes perfectly sense.

The changes we detected in the cerebellum were mainly in the expected direction, considering its close relationship with movement control and learning. Once learned, the skilled movements remain coded in the cerebellar memory cells for a long time. The cerebellum then provides speed, complexity, variety, stereotypy, and automaticity of the motor response so that one does not have to think consciously about the movement. It has been suggested that the learned programs are stored within the cerebellar cortex and that the memory capacity for storage is proportionate to the number of granule cells (Thach, 1998; Boyden et al., 2004). Our study revealed a major GM expansion in the superficial layers of the cerebellum, which could be perhaps due to the increment in the capacity to store complex movement-related memories in ballet dancers. On the other hand, we observed significantly smaller GM volumes in slightly deeper cerebellar structures of the ballet dancers, parts that are known to be involved in the control of limb movements, which can be interpreted in terms of automaticity/stereotypy.

One earlier study on ballet dancers has shown that the right hemispheric visual dominance is particularly useful for postural control in complex equilibrium conditions (Golomer et al., 2010). In our study, we could also observe differences that were mainly localized on the right side of the brain.

Main between-group differences in the thalamus were observed for its posterior part, and only on the right side. Earlier studies have proposed a functional importance of the posterolateral thalamus as a unique relay station for vestibular input to the cortex, but also the dominance of the right hemisphere in right-handedness, and of ipsilateral ascending pathways (Dieterich et al., 2005). Multiple thalamic nuclei are involved in vestibular processing, as well as somatosensory and visual, and this may explain the enlarged thalamus in the dancers (Morel et al., 1997; Lopez and Blanke, 2011).

Possible neurobiological mechanisms of the observed GM differences could be neurogenesis, synaptogenesis, hypertrophy of glia cells, and angiogenesis (Zatorre et al., 2012). The generation of new cells within the confines of our findings can only be expected for the hippocampus, but not for other areas of the ballet dancers’ brain (Bhardwaj et al., 2006). Hippocampal neurogenesis in the adult has also been undermined somewhat by a recent publication (Sorrells et al., 2018). Instead, the observed GM increments are presumably based on the sensory experience which drives the formation and elimination of synapses and these changes might underlie adaptive remodeling of neural circuits (Trachtenberg et al., 2002). Importantly, animal studies have reported that motor learning of complex and acrobatic skills, and not repetitive use of synapses during simple physical exercise, generates new synapses in the cerebellar cortex (Black et al., 1990). In contrast, simple exercise leads to a greater density of blood vessels in the cerebellum.

The main limitation of our study is its cross-sectional nature, whereby no causal relationship for the effects observed can be established. Previous studies have, however, shown that training-induced neuroplastic adaptations are actually sport-specific rather than just sport-general. For instance, GM volumes in the hand representations are increased in handball players compared with ballet dancers, whereas GM volumes in the foot representations are increased in ballet dancers compared with handball players (Meier et al., 2016). Similarly, differences were observed between martial artists and endurance athletes (Schlaffke et al., 2014), but also musicians and non-musicians (Gaser and Schlaug, 2003), world class gymnasts (Huang et al., 2015), golfers with various skill levels and non-golfers (Jäncke et al., 2009), and sprinters and endurance runners (Wenzel et al., 2014), who differ in GM volumes of the anterior cerebellar lobe, and in vermian lobules compared to basketball players (Park et al., 2009). Considering the cross-sectional nature of these studies, some of these multiregional differences may be attributable to innate predisposition. Additionally, GM alterations in the cortex can occur as early as after 7 days of training (Driemeyer et al., 2008), and the temporal dynamics of the structural changes may lead to a partial or complete loss of these effects (May and Gaser, 2006; Taubert et al., 2010). Nevertheless, many researchers believe the neuroanatomical effects found in professional athletes may represent structural adaptations in response to long-term skill acquisition and the repetitive rehearsal of those skills. Some studies have shown that visual input is of a great importance for ballet dancers (Hugel et al., 1999; Perrin et al., 2002; Golomer et al., 2009), in order to successfully maintain balance and perform complex movements; however, we could not find any structural differences in visual cortex.

## Conclusion

Our study demonstrated significant differences between professional ballet dancers and non-dancers in their neuroanatomy as well as in their abilities pertained to balancing and non-visual wayfinding. Some anticipated brain regions revealed structural alterations in professional ballet dancers, such as the cerebellum, the cingulate motor cortex, the posterior thalamus, as well as the posterior hippocampus and parahippocampus. In accordance with these structural observations, the dancers were able to better maintain balance, both in static and dynamic conditions, and to more accurately complete the triangular path both in the walking (somatosensory and vestibular) and particularly in the wheelchair (vestibular only) condition. We conclude that intensive life-long ballet training is presumably the main cause for the differences detected in our study.

## Author Contributions

MD organized the study, analyzed the data, and wrote and reviewed the paper. RS collected the data and wrote the paper. MT analyzed the data and reviewed the paper. PM reviewed the paper. AH organized the study and reviewed the paper. NM organized the study, and wrote and reviewed the paper.

## Conflict of Interest Statement

The authors declare that the research was conducted in the absence of any commercial or financial relationships that could be construed as a potential conflict of interest.

## Footnotes

1 http://www.alivelearn.net/xjview
